# Heavy Metal Contamination in *Oryza sativa* L. at the Eastern Region of Malaysia and Its Risk Assessment

**DOI:** 10.3390/ijerph19020739

**Published:** 2022-01-10

**Authors:** Nur Syahirah Zulkafflee, Nurul Adillah Mohd Redzuan, Sara Nematbakhsh, Jinap Selamat, Mohd Razi Ismail, Sarva Mangala Praveena, Soo Yee Lee, Ahmad Faizal Abdull Razis

**Affiliations:** 1 Department of Food Science, Faculty of Food Science and Technology, Universiti Putra Malaysia, Serdang 43400, Malaysia; nursyahirahzulkafflee@gmail.com (N.S.Z.); dillaredzuan94@gmail.com (N.A.M.R.); sjinap@gmail.com (J.S.); 2 Laboratory of Food Safety and Food Integrity, Institute of Tropical Agriculture and Food Security, Universiti Putra Malaysia, Serdang 43400, Malaysia; saranematbakhsh@gmail.com (S.N.); smpraveena@upm.edu.my (S.M.P.); 3 Laboratory of Climate-Smart Food Crop Production, Institute of Tropical Agriculture and Food Security, Universiti Putra Malaysia, Serdang 43400, Malaysia; razi@upm.edu.my; 4 Department of Environmental and Occupational Health, Faculty of Medicine and Health Sciences, Universiti Putra Malaysia, Serdang 43400, Malaysia; 5 Natural Medicines and Products Research Laboratory, Institute of Bioscience, Universiti Putra Malaysia, Serdang 43400, Malaysia; leesooyee@upm.edu.my

**Keywords:** heavy metals, enrichment factor, translocation factor, health risk assessment

## Abstract

Paddy plants tend to accumulate heavy metals from both natural and anthropogenic sources, and this poses adverse risks to human health. The objective of this study was to investigate heavy metal contamination in paddy plants in Kelantan, Malaysia, and its health risk assessment. The bioaccumulation of heavy metals was studied by means of enrichment (EF) and translocation factors (TF). The health risk assessment was performed based on USEPA guidelines. The EF for heavy metals in the studied areas was in the descending order of Cu > As > Cr > Cd > Pb. Meanwhile, Cr and Pb exhibited higher TF values from stem to grain compared with the others. The combined hazard index (HI) resulting from five heavy metals exceeded the acceptable limit (HI >1). The lifetime cancer risk, in both adult and children, was beyond the acceptable limit (10^−4^) and mainly resulted from exposure. The total cancer risk (CRt) due to simultaneous exposures to multiple carcinogenic elements also exceeded 10^−4^. In conclusion, intake of heavy metal through rice ingestion is likely to cause both non-carcinogenic and carcinogenic health risks. Further research is required to investigate the extent of heavy metal contamination in agricultural soils and, moreover, to establish human exposure as a result of rice consumption.

## 1. Introduction

Kelantan, also known as the “Land of Lightning”, is a state located at the northeast region of Peninsular Malaysia. It is an agrarian state well known for its green paddy fields, rustic fishing villages and casuarina-lined beaches. The Kelantan River is the main river in Kelantan, where it dominates the fertile coastal plains and defines the geography of the state. The Kelantan River valley is a fertile land for the plantation of *Oryza sativa* L. (rice), besides being rich in hardwoods and rubber, and lush with tropical fruits.

Being one of the most consumed foods, rice supplies nearly 50% of the daily caloric intake of people worldwide [1,2,3,4]. The global rice consumption is predominated by Asian countries, as rice is the major staple food and the daily consumption of rice among Asian populations has increased up to 0.5 kg per capita, on a dry weight basis [5]. In Malaysia, rice is the third most important agricultural crop [6,7,8,9]. To meet the increased demand of rice, rice producers have used escalating amounts of synthetic fertilisers and other agrochemicals containing heavy metals such as arsenic, cadmium and lead to increase crop yield and maximise profit. [10,11]. This has become a great concern because the paddy fields tend to be contaminated by heavy metals due to the non-biodegradability of heavy metals. Heavy metals have been categorised as one of the major chemical hazards due to the potential risk they pose to the environment and to human health. Heavy metals (under natural conditions) can exist as both organic and inorganic species [12].

Modern agricultural practices such as non-selective application of agrochemicals such as pesticides and fertilisers in addition to mechanical cultivation may possibly contaminate the agricultural soils with essential and nonessential heavy metals [13,14]. The adsorption and accumulation of heavy metals in the layer of soil are probably due to relatively high organic matter, which has a direct effect on the cation exchange capacity and buffer capacity, as well as the retention of heavy metals [15]. Moreover, different plants, as well as different parts of the plants, have different capacities in the absorption and accumulation of heavy metals including the metal uptake and translocation between plant species and even between cultivars of the same plant species [15,16,17,18,19,20,21,22].

The transfer of heavy metals from paddy plant to the human body, hence posing a danger to human health, is through the consumption of rice grain, which is the edible component of the paddy plant [23]. Since rice is consumed as a staple food by the majority of the world’s population, including Malaysia, it is crucial to assess the levels of heavy metals in paddy soils and paddy plants, and to elucidate the mechanisms of this heavy metal uptake, as continuous intake of contaminated rice poses adverse health risks. Known as the Rice Bowl of Malaysia, Kedah is the major rice-producing state in Malaysia and many studies concerning heavy metals have focused on the plantation areas in this state [7,17,24,25,26]. In addition, the other states known for paddy plantation, including Perlis, Sabah and Selangor, have also been studied for heavy metal contamination in paddy soil and plants [26,27,28,29]. However, to date, the safety of rice in Kelantan in relation to heavy metal contamination is still not well documented, and the information on heavy metal accumulation as defined by the enrichment factor and translocation factor is also lacking. Moreover, the health risk associated with these heavy metals via rice consumption among the local population has not been systematically addressed.

Therefore, this study aimed to assess the accumulation of heavy metals (Pb, Cd, Cu, Cr and As) in paddy plants cultivated in Kelantan, and to assess the health risk associated with these heavy metals through rice consumption. Paddy fields in three different districts, namely Kota Bharu, Pasir Mas and Pasir Puteh, were selected for sampling of paddy soils and plants. The sampling sites are far away from the industrial and residential areas, and the river (Golok River) providing the water source for irrigation has been categorised as clean and unpolluted (Class II) by the Department of Environment (DOE) Malaysia [30]. Thus, presence of heavy metals in paddy soils may derived from anthropogenic sources, mainly by application of agrochemicals. Findings from this present study provide information regarding heavy metal contamination in soil and paddy plants in Kelantan as well as the associated potential health risks. In view of the detrimental effects in human health due to dietary intake of heavy metals through rice consumption, these findings are deemed crucial as they reflect the safety of rice cultivated in the studied areas in Kelantan and justify the necessity of control measures for the management of heavy metal contamination in Kelantan as well as in Malaysia, especially the usage of agrochemicals in paddy plantation.

## 2. Materials and Methods

### 2.1. Chemical Reagents and Materials

Chemicals used for the extraction of heavy metals from soil and paddy plants, including ammonium acetate, 69% nitric acid and 60% perchloric acid, were supplied by Merck (Darmstadt, Germany). The 0.45 µm syringe filter was supplied by Merck Millipore (Guyancourt, France).

### 2.2. Sampling Sites

**Figure 1:**
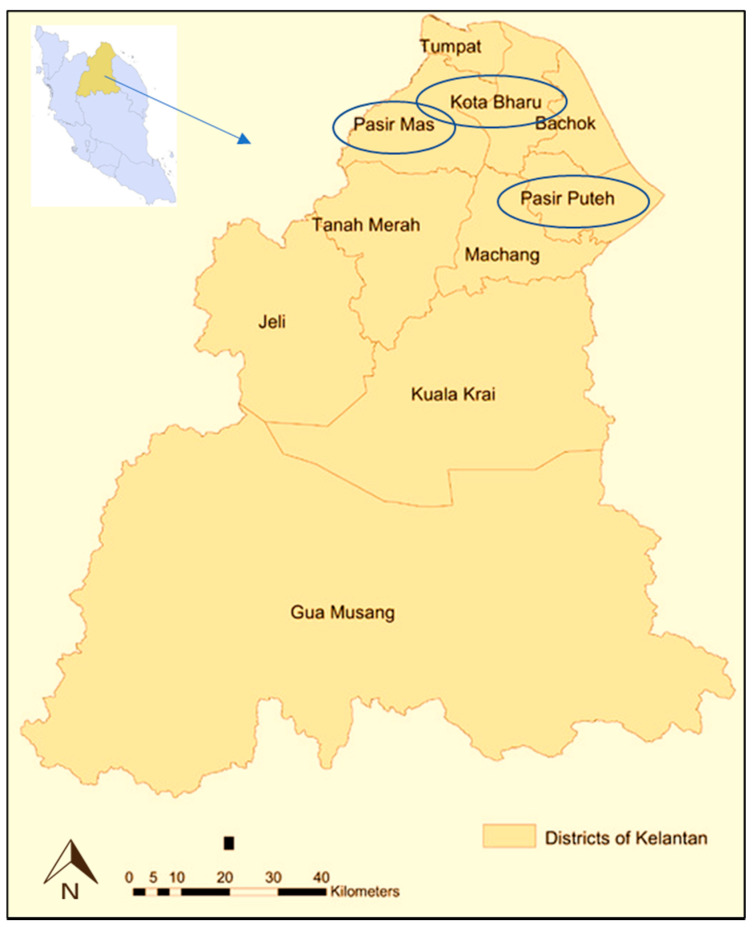
Map showing the sampling areas at Kelantan, Malaysia Eastern region.

Kota Bharu (6.1248° N, 102.2544° E), Pasir Mas (6.0424° N, 102.1428° E) and Pasir Puteh (5.8362° N, 102.4077° E) were selected as the sampling sites in Kelantan, which represents the north-east of Peninsular Malaysia (Figure 1). The reason for choosing Kelantan for sampling is that it is one of the important rice-producing states in Malaysia. Paddy fields in the Kelantan are coordinated by the Kemubu Agricultural Development Authority (KADA). This authority oversees the irrigation system and aids the farmers in the production. The Kemubu paddy field is recognised as the second largest paddy plantation area in Malaysia, after the Kedah-Perlis paddy field area. In this study, three paddy plantation areas, which plant the MR220 CL2 variety, were selected for sampling. At each sampling area, three plots of 1000 m^2^ were selected and the number of samplings was thrice (*n* = 3) at each plot. Sampling was conducted in October 2018 when the paddy plant in the field was fully mature (growth stage #8, code 89). For soil samples, the soil was obtained from the surrounding area of the paddy plant roots using a hand-operated soil auger, at 0 to 30 cm depth. For paddy plant samples, a number of paddy plants (consisting of root, stem, leaf and grain) were uprooted carefully from each plot and kept in clean plastic bags [6]. All the samples were labelled appropriately, according to the sampling sites, namely KBP1, KBP2, KBP3 for Kota Bharu plot 1, 2 and 3, respectively; PMP1, PMP2, PMP3 for Pasir Mas plot 1, 2 and 3, respectively; and PPP1, PPP2 and PPP3 for Pasir Puteh plot 1, 2 and 3, respectively.

### 2.3. Extraction of Heavy Metals from Paddy Soil

Soil samples were processed as described [6], with slight modifications. The samples, prepared in triplicate from each plot area, were dried at room temperature in the laboratory. The dried soil was powdered and passed through a mesh sieve (No. 60, 250 µm, Humboldt, Elgin, IL, USA). Ammonium acetate (NH_4_CH_3_OO, 1 M, pH 7) was then used for the extraction of heavy metals from the soil samples. Briefly, powdered soil (10 g) was mixed with NH_4_CH_3_OO (50 mL), followed by shaking for 90 min. Then, the mixture was centrifugated at 3000 rpm for 30 min and the supernatant was filtered through a 0.45 µm Millipore filter paper before being subjected to analysis.

### 2.4. Extraction of Heavy Metals from Paddy Plants

The collected paddy plants were separated according to their different parts, namely the grain, leaf, stem and root. After cleaning with running tap water and rinsing with distilled water, the separated samples were dried in an oven (Memmert, Schwabach, Germany) at 35 °C. The drying process completed while the samples had reached constant weight. Dried leaf, stem and root were ground using a blender whereas the dried grain was crushed using pestle and mortar. All powdered samples were sieved through a 250 µm mesh sieve (No. 60 mesh sieve, Humboldt, Elgin, IL, USA). Three replications were prepared for each sample collected from each sampling plot. Heavy metals in the different parts of paddy plant (root, stem, leaf and grain) were extracted employing acid digestion method [6]. A gram of sample was weighed, in which 10 mL of 69% nitric acid was added using an adjustable 10 mL single-channel pipette (Eppendorf Research, Hamburg, Germany). The mixture was then heated at 60 to 80 °C on a hot plate (Heidolph MR 3001, Heidolph Instruments, Schwabach, Germany) to allow the digestion to take place until brown gas was produced. Periodically, 60% perchloric acid (5 mL) was added until the mixture was clear. After cooling down, 20 mL of distilled water was transferred to the mixture, and subsequent filtration was performed using a 0.45 µm Millipore filter paper and syringe. The filtrate was finally made up to 50 mL with distilled water in a 50 mL volumetric flask.

### 2.5. Quantification of Heavy Metals

Heavy metals extracted from paddy plants and soils were quantified using inductively coupled plasma mass spectrometry (ICP-MS) (ELAN DRC-e, PerkinElmer, Waltham, MA, USA), and the results were expressed in mg/kg. The elements analysed in this study included lead (Pb), cadmium (Cd), copper (Cu), chromium (Cr) and arsenic (As). A total of 10% nitric acid (HNO_3_) was employed to soak all the lab equipment to maintain the quality. Moreover, to ensure the reliability of measurements, each soil and paddy plant sample was analysed in triplicate, together with Certified Reference Material (CRM) IRMM 804 (Institute for Reference Materials and Measurements, Geel, Belgium) in the range of 90.5–102.4%. A blank sample and the standard solution were also analysed after every 10 samples to ensure no carryover of analytes from the previous samples.

### 2.6. Enrichment Factor (EF) and Translocation Factor (TF)

The enrichment (*EF*) and translocation factors (*TF*) were analysed using Equations (1) and (2), respectively [31,32]. The *EF* reveals the absorption of heavy metals from soil to paddy grain, the edible portion of the paddy plant, while the *TF* refers to the transport of heavy metals from soil to the different parts of the paddy plant (root, stem and grain).

(1) $$Enrichment\;factor\;\left(EF\right)=\frac{Cp}{Cs}$$

where *Cp* and *Cs* denote the heavy metal concentration in the paddy grain and soil, respectively.

(2) $$Translocation\;Factor\;\left(TF\right)=\frac{Cs}{Cr}$$

where *Cs* represents the heavy metal concentration in the plant parts and *Cr* the heavy metal concentration in soil, root or stem.

### 2.7. Health Risk Assessment (HRA) of Heavy Metals

#### 2.7.1. Average Daily Dose (ADD)

Average daily dose (ADD) is used to quantify the oral exposure to heavy metals via rice consumption for a specific period and the results were expressed in mg/kg.day, which is a daily dose per unit body weight [13]. ADD was determined employing Equation (3):

(3) $$\mathrm{ADD}\;\left(\mathrm{mg}/\mathrm{kg}\cdot \mathrm{day}\right)=\frac{\mathrm{Crice}\times \mathrm{IR}\times \mathrm{ED}\times \mathrm{ExF}}{\mathrm{Bw}\;\times \mathrm{AT}}$$

The average concentrations of heavy metals in rice (Crice) were obtained from the ICP-MS analysis in this current study. The average rice ingestion rate (IR), exposure frequency (ExF), exposure duration (ED) and body weight (Bw) values for Malaysians were taken from the reports of Department of Statistics Malaysia [33,34,35]. For adults, the IR, ExF, ED and BW were 0.6 kg/day, 365 days/year, 74 years and 62.65 kg, respectively [34,35]. Meanwhile, the average IR, ExF, ED and BW for children were 0.198 kg/day, 365 days/year, 74 years and 19.5 kg, respectively [34,35]. The IR from the Chinese population [35] was used since there is no available data for Malaysian children. The averaging time (AT) of non-carcinogenic was derived from ED × 365 days based on USEPA.

#### 2.7.2. Hazard Quotient (HQ)

HQ was calculated by using Equation (4) based on USEPA as an indicator of the non-carcinogenic risk in the inhabitants of the Kelantan areas following consumption of rice. HQ is defined as the ratio of ADD to the reference dose (RfD) [36,37]. The equation for calculating HQ is:

(4) $$\mathrm{HQ}=\frac{\mathrm{ADD}}{\mathrm{RfD}}$$

where RfD (Table 1) is the reference maximum allowed human doses of the heavy metals via daily exposure [38].

#### 2.7.3. Lifetime Cancer Risk (LCR)

LCR was calculated by multiplying the ADD (mg/kg.day) across a lifetime with the cancer slope factor (CSF), as shown in Equation (5). The averaging time (AT) in calculating ADD for carcinogenic risk was 25,550 days based on USEPA. LCR is evaluated as the incremental probability of a human getting cancer during the course of a lifetime [38,39]. As an example, an LCR of 10^−4^ implies the probability that 1 in 10,000 individuals will develop cancer. The LCR of local people in Kelantan areas resulted from exposure to potential carcinogen was calculated using Equation (5) [40,41,42]:

LCR = ADD (mg/kg/day) × CSF (mg/kg/day)(5)

The combination effect of multiple carcinogenic elements was reported as total cancer risk (CRt) by summing up the values of LCR from individual carcinogen, as shown Equation (6):

(6) $$\mathrm{CRt}=\sum^{\;}\mathrm{LCR}$$

The risks in the range of 1.0 × 10^−6^ to 1.0 × 10^−4^ for LCR and CRt values are acceptable [42,43]. Cr, Cd and As were classified as carcinogenic elements, whereas Pb was regarded as possibly carcinogenic to humans. Cu has not been classified as a carcinogenic element, as defined by the International Agency for Research on Cancer.

### 2.8. Statistical Analysis

All experiments were conducted in triplicate and the concentrations of heavy metals are reported as mean ± standard deviation (SD). Data were evaluated statistically using MINITAB software (Version 17.0, Minitab Inc., State College, PA, USA) using general linear model (GLM) and Tukey’s pairwise comparison by considering the probability level of *p* < 0.05 as statistically significant. Moreover, the same software was used to conduct a principal component analysis (PCA) to evaluate the classification of sampling sites based on the level of heavy metals in paddy soil and different parts of paddy plant.

## 3. Results and Discussion

### 3.1. Heavy Metal Concentration in Paddy Soil

Heavy metal concentrations in paddy soils collected from Kota Bharu, Pasir Mas and Pasir Puteh are shown in Table 2. Unlike the previous studies which reported the relatively low content of arsenic (As) in paddy soil in the Kedah and Sabah plantation areas [17,27], As exhibited the highest concentration in all studied areas in the current study, followed by lead (Pb), copper (Cu), chromium (Cr) and cadmium (Cd). However, the concentration of the studied elements in paddy soils collected from all studied areas was below the maximum permitted level mentioned in Chinese Environmental Quality Standard for Soils, grade II [40] and European standard agriculture soils [41]. The relatively low accumulation of Cd in paddy soils was in line with the previous reports in Malaysia where the value was less than 0.2 mg/kg in average (at ratio 1:1; root to soil) [9,20,29]. As shown in Table 2, concentration of Pb, Cd, and Cr is not significantly different (*p* > 0.05) among all studied sampling sites. Meanwhile, PPP sampling sites had the significantly highest concentration of As in comparison with the two other sampling sites, and the lowest As concentration was observed in the KBP sampling sites. The high content of As in paddy soils, as the result of anthropogenic contamination, will increase the level of As in rice since diffusion from the soil is the major source [44]. Moreover, paddy soil in the PMP sites had significantly higher Cu concentrations compared with PPP sites, whereas there was no significant variation in Cu concentration between KBP sites and other two sampling sites.

### 3.2. Heavy Metal Concentration in Paddy Plants

The mean and standard deviation of arsenic concentrations in paddy plant parts (root, stem, leaf and grain) collected from Kelantan paddy plantation areas are shown in Table 3. As can be seen, the concentration of As in the root and grain of paddy plants had almost the same value among all three sampling sites. The significant higher level of As concentration can be found in stem of paddy plants located in the KBP sampling sites and paddy leaf located in PPP sites. These parts are highly accumulated with As, indicating high availability of As in the soil in addition to its mobility within the plants [24]; concentration may be also influenced by various anthropogenic practices such as farming tractors, chemical fertiliser and pesticide usage [29]. The arsenic distribution from the root to grain of the paddy plant from the three plots (PPP1, PPP2, PPP3) ranged from 0.078 to 0.087 mg/kg for As concentration, in which the concentration of As in PPP sites was highest in leaf and grain of paddy plants. In fact, the concentration of As in the paddy leaf of plants from the Pasir Puteh was markedly higher than other parts. These results were inconsistent with the previous studies whereby the roots accumulated highest contents of heavy metals [29,32,45]. Arsenic accumulation is closely associated with the presence of iron plagues on the root surface [46]. The dynamics of As in the soil region around the paddy roots of may play a key role in the oxidation of arsenite to arsenate by the iron plaque or the release of oxygen from paddy roots [47]. Meanwhile, As content in the grain of paddy plants located in PPP sites is much higher than the other studied heavy metals, and this observation is consistent with data for heavy metals accumulation in paddy plants in China, Turkey and Taiwan [48,49,50]. It is known that inorganic As (iAs) that is present in rice is the most toxic As species. Inorganic As is the dominant species in Asian and European rice, comprising about 30 to 100% of total As [5].

The mean concentrations of Cd in the different parts of paddy plant sampled from different areas in Kelantan are presented in Table 4. Interestingly, there was no statistically significant differences in Cd content in all parts of paddy plants (root, stem, leaf and grain) among all the three sampling sites. The Cd levels in the various parts of paddy plants were equally low for all sampling areas. In fact, Cd is the rare heavy metal in the paddy plants; specifically, the level of Cd was the lowest in the grains than in the other parts. The results of this current study were dissimilar with previous reports, in which Cd is readily absorbed by plants and distributed to the different parts, and lend support to the view that Cd is not readily absorbed by the plants and distributed to the tissues [51,52]. The presence of Cd is usually characterised by oxidative stress [53,54].

Table 5 displays the concentrations of lead in paddy plant parts sampled from different paddy plantation areas in Kelantan. As observed, there was no significant difference regarding lead concentration in grain and root of paddy plants among all sampling sites. Moreover, the highest lead content was in paddy stem of KPB sites and paddy leaf in PPP sampling sites. The concentration of Cd and Pb in rice grain collected from all studied sites was low, in contrast to previous studies that reported their high accumulation in food crops [34,55]. The discrepancy may be due to the different local geology and agricultural practices.

Table 6 shows the mean and standard deviation of copper concentrations in various parts of paddy plants sampled from Kelantan. Based on the results, there were no significant differences (*p* < 0.05) in Cu content of paddy root and stem among three sampling sites. However, markedly high Cu content was found in the paddy leaf and stem located in KBP sites compared with other sampling sites. Overall, the significantly highest and lowest (*p* < 0.05) Cu concentration was found in the leaf and grain of paddy plants located in KBP sampling sites, respectively. Micronutrients such as Fe, Mn, Zn and Cu are encountered in paddy plants since they are essential for several enzymatic activities and play significant functions in plant growth and photosynthesis [13]. In all samples collected from the Kota Bharu Plot (KBP2) (data not shown), Cu content was the highest in the stem compared with other parts, which is in accordance with previous studies [29,45]. The distribution of Cu is greater in the root since only 10% of the absorbed Cu was translocated in the stem [56].

Table 7 shows the mean and standard deviation of chromium concentrations in various parts of paddy plants sampled from Kelantan. The level of Cr content was in the same range in root, stem, and grain of paddy plants in all three sampling sites. The paddy plants available in PMP site had the lowest Cr concentration in their leaf compared with other sampling sites. The maximum permitted level of Cr in rice grain has not been defined but its content appears to be higher compared with the shoot part (stem and leaf). Kabata-Pendias and Pendias [57] recommended the safe level for Cr in paddy plants to be 30 mg/kg, while in Japan, the proposed acceptable limit is 35 mg/kg for Cr in the upper parts of the paddy plants. In the present study, Cr was found mostly in the grain compared with other parts of the paddy, which contradicts previous findings where the roots were reported to accumulate Cr more effectively than the stem, leaf and grain [32,45]. This was attributed to a redox reaction between chromium (III) and carboxylic functional groups in the plants encouraging the translocation of Cr from the root to the shoot part [58]. However, findings from the present study do not support the translocation of Cr from the root to the shoot. The elevated level of Cr in the rice grain from the Pasir Puteh area may be a consequence of anthropogenic contamination in the vicinity of the paddy field. Although Cr is considered an essential element, excessive levels in food or plants may cause toxicity to animals and humans [24].

Overall, the accumulation of heavy metals in rice is not solely dependent on their concentration in soil but maybe also be influenced by the physicochemical properties of the soil [59]. The pH can affect the sorption and desorption of heavy metals towards other soil components, affecting the ability to transfer metals [60]. A rise in acidity promotes the movement of heavy metals from soil to rice. From the present finding, it is evident that all analysed heavy metals are accumulated in all parts of the paddy plant at different degrees. Overall, the heavy metals accumulated the most in the root in comparison with stem, leaf and grain, in agreement with previous findings concerning the distribution of heavy metals in paddy plants [29,45]. The root plays an important role as a barrier for metal transferability, hence providing protective effect to the stem and grain against the contamination [61]. Most of the heavy metals that are absorbed by paddy plants accumulate in the root due to its high metabolic rate, resulting in relatively low levels in the stem and leaf [62].

### 3.3. Enrichment Factor (EF)

**Figure 2:**
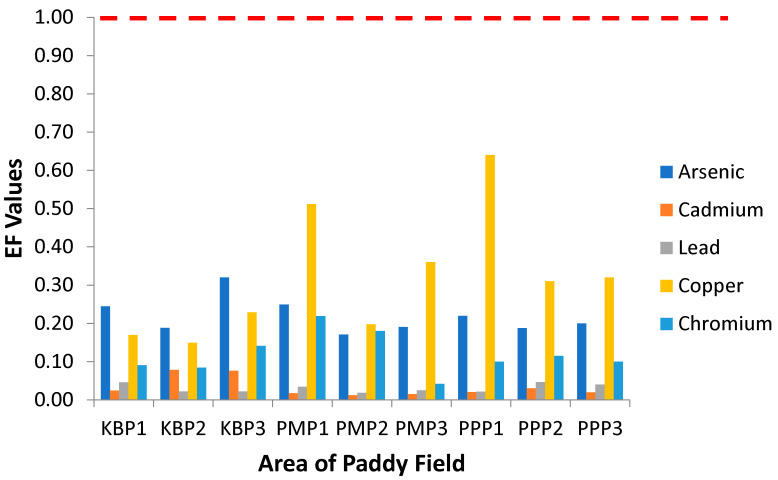
Enrichment factor (EF) of the heavy metals in the different sampling sites. KBP1: Kota Bharu Plot 1, KBP2: Kota Bharu Plot 2, KBP3: Kota Bharu Plot 3, PMP1: Pasir Mas Plot 1, PMP2: Pasir Mas Plot 2, PMP3: Pasir Mas Plot 3, PPP1: Pasir Puteh Plot 1, PPP2: Pasir Puteh Plot 2, and PPP3: Pasir Puteh Plot 3.

Food chain is an important route for human exposure to soil contaminants [34]. Inorganic pollution possibly increases when the plants are able to accumulate trace elements from anthropogenic sources rendering them passive biomonitors [63]. In the present study, the enrichment factor (EF) of all studied metals was less than 1, in accordance with previous findings which stated that paddy plants absorb but do not accumulate heavy metals and thus having low metal bioavailability [25,29,32]. Figure 2 presents the enrichment factor of heavy metals from soil to paddy grain, the edible part of paddy plants, collected from Kota Bharu, Pasir Mas and Pasir Puteh plantation areas. The EF values for all studied heavy metals were found to be lower than 1, ranging from 0.06 to 0.51, 0.17 to 0.32, 0.04 to 0.22, 0.01 to 0.08 and 0.02 to 0.05 for Cu, As, Cr, Cd and Pb, respectively. The EF for heavy metals in the studied areas was in the descending order of Cu > As > Cr > Cd > Pb. The EF ≤ 1 indicates only absorption, but no accumulation of heavy metals by the plant. Meanwhile, EF > 1 indicates the plant accumulates the heavy metals. Among the studied metals, EF values in paddy plants from the Pasir Puteh Plot 1 (PPP1) were found to be the highest for Cu, whereas in the PMP2, the lowest EF values for Cd. The latter does not lend support to previous studies where Cd was found to deposit in rice grain from contaminated soil and hence posing risks to human health [38,64]. The difference in Cd concentration between soil and grain was found to be relatively low in the present study. The high level of Pb in soil does not lead to its buildup in rice grain, indicating the inability of the plant to accumulate this metal. The absorption of heavy metals from the soil is dependent on many factors such as plant species, the level of metal in the soil and bioavailability of the metal which is affected by both the physical and chemical properties of the soil [65].

### 3.4. Principal Compenent Analysis (PCA)

**Figure 3:**
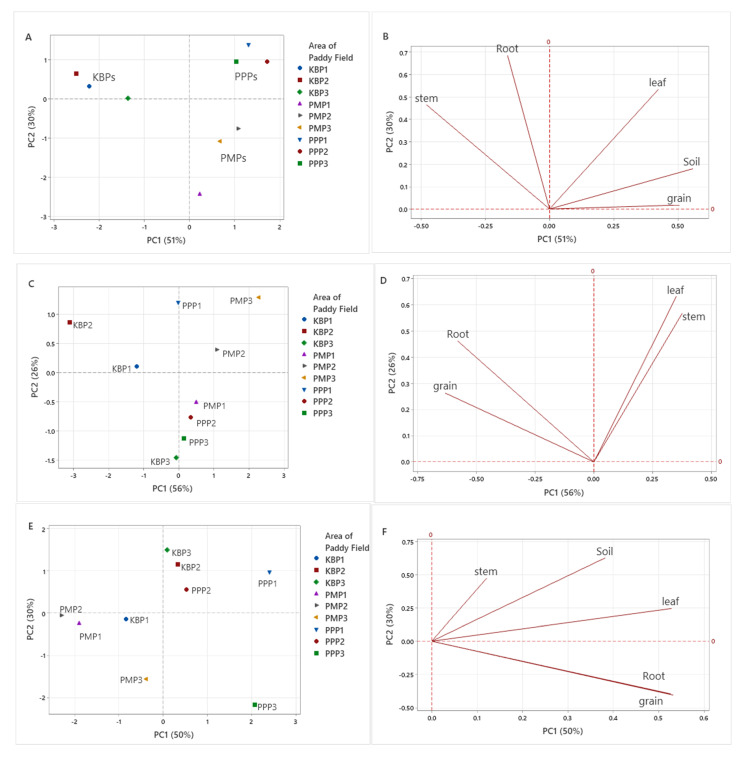

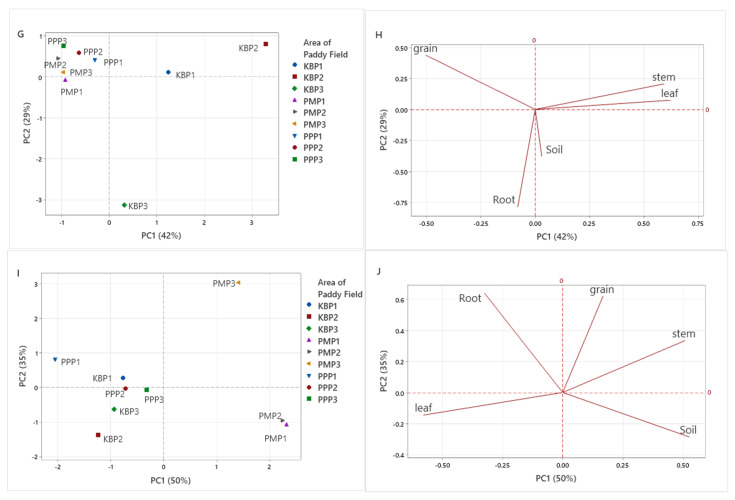
PCA score and loading plots showing the clustering pattern of different sampling sites based on the heavy metal concentration in paddy soil and paddy plant parts. (**A**,**B**) Score and loading plots of arsenic concentration, (**C**,**D**) cadmium concentration, (**E**,**F**) lead concentration, (**G**,**H**) copper concentration and (**I**,**J**) chromium concentration in different sampling sites.

Principal component analysis (PCA) is a statistical tool used to obtain a comprehensive idea of the relationship between different variables. In this study, PCA was performed to gain knowledge about the correlation between different sampling areas and the concentration of heavy metals in paddy soil and paddy plant parts. Figure 3A–J shows the PCA for the relationship between different sampling areas and the concentration of the heavy metals. Accordingly, in Figure 3A, two principal components (PC) with eigenvalues >1 were extracted to best discriminate the different sampling area based on the arsenic concentration, in which the PC1 and PC2 described 51% and 30% of the total variation in the data, respectively. From this figure, it can be observed that each sampling sites (KBP, PMP, PPP) had specific clustering trends (95% confidence interval). The sampling sites for PMP and PPP can be significantly distinguished from sampling site KBP by virtue of the signs of the PC1 scores, in which the PMP and PPP sampling sites were at the positive score of PC1, and showed the higher concentration of arsenic in soil, grain and leaf compared with the paddy plants located in the KBP sampling site, which is at the negative side of the PC1. Moreover, based on Figure 3B, paddy fields located in KBP had higher concentration of arsenic in their stem and root.

Regarding to cadmium concentration, Figure 3C showed the close separation of sampling site, KBP from two other sampling sites based on the PC1, which accounted for the 56% of the variance. Therefore, based on Figure 3C,D, it can be concluded that paddy fields located in the PMP and PPP sampling sites had higher concentration of cadmium in their leaf and stem, with high positive loading on PC1, compared with the paddy fields located in KBP, more precisely KBP1 and KBP2, which had higher concentration of cadmium in root and grain. Since all three sampling areas (KBP, PMP and PPP) shared the same concentration of cadmium in paddy soil (0.012 mg/kg), discrimination of the sampling sites based on the soil Cd-content data was unable to be analysed.

In terms of the lead concentration, Figure 3E,F indicated that the paddy fields located in the PMP significantly separated from paddy plants in KBP and PPP, in the virtue of PC1 (accounted for 50% of variance). As can be seen in Figure 3F, paddy fields in KPB2, KBP3, PPP1 and PPP2 sites which was located on the positive site of PC1, had higher concentrations of lead in their leaf, soil and stem, while the concentration of lead in root and grain of paddy fields located in PPP3 was higher compared with other paddy plants sites. Therefore, the paddy fields in the PMP sites had the lowest lead concentration in paddy soil and plants.

Regarding to copper concentration, Figure 3G,H showed that the PC1 (42%) was responsible for the separation of paddy fields located in KBP sites from other locations based on the copper concentration in paddy soil and different paddy parts. Moreover, based on Figure 3H and Table 6, the paddy plants located in KBP sites had higher copper concentrations in stem and leaf parts. While the paddy plants located in the PMP and PPP sites had higher copper concentration in the grain compared with KBP sites. Furthermore, the KBP3 site which is located on negative area of PC2 (29%) had high value for copper concentration in the root compared with other sampling sites.

The investigation about chromium concentration in paddy soil and parts in nine different paddy plant sites (Figure 3I,J) revealed that paddy plants located in the PMP sites significantly separated from paddy plants available in other sites including KBPs and PPPs. The PMP sites which were located on the positive area of PC1 had higher values for chromium concentration in grain and stem and soil, in which PMP1 and PMP2 had the highest chromium concentration in the paddy soil, and PMP3 had the highest chromium concentration in stem and grain compared with other sampling sites. On the other hand, the paddy plants located in the KBP and PPP sites had higher chromium concentration in the paddy leaf part compared with paddy plants in PMP sites. In fact, based on Table 7, the lowest chromium concentration was found in the paddy leaf located in the PMP sampling site.

### 3.5. Translocation Factor (TF)

**Figure 4:**
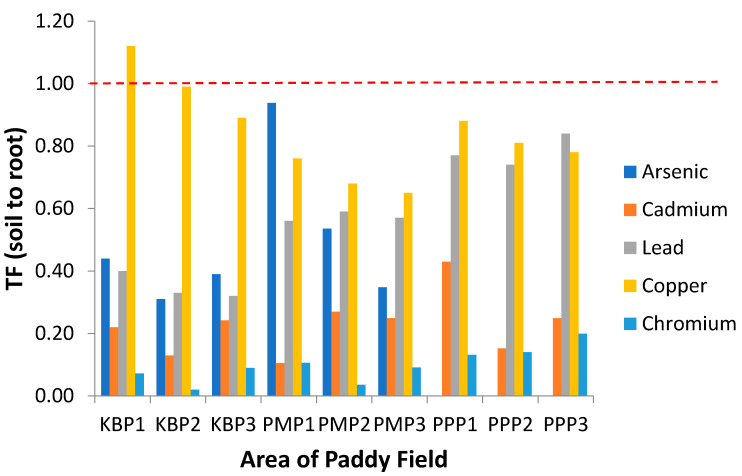
Translocation factor of the heavy metals from soil to root (TF_Soil_) at different sampling sites. KBP1: Kota Bharu Plot 1, KBP2: Kota Bharu Plot 2, KBP3: Kota Bharu Plot 3, PMP1: Pasir Mas Plot 1, PMP2: Pasir Mas Plot 2, PMP3: Pasir Mas Plot 3, PPP1: Pasir Puteh Plot 1, PPP2: Pasir Puteh Plot 2, and PPP3: Pasir Puteh Plot 3.

**Figure 5:**
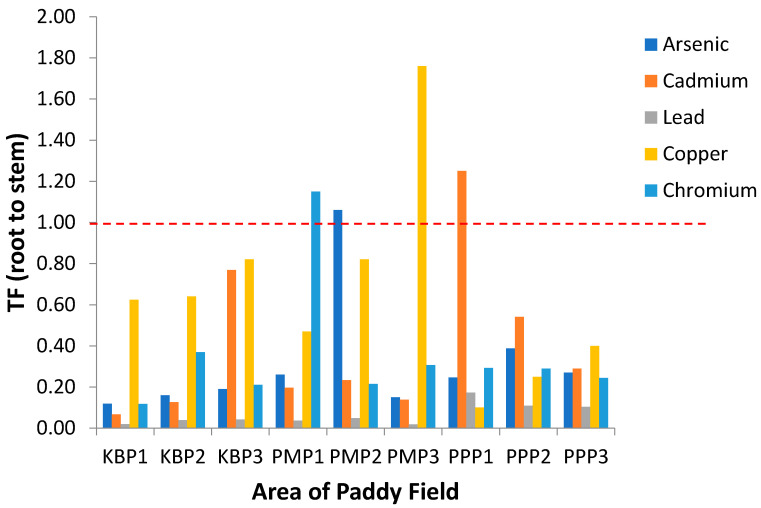
Translocation factor of the heavy metals from root to stem (TF_Root_) at different sampling sites. KBP1: Kota Bharu Plot 1, KBP2: Kota Bharu Plot 2, KBP3: Kota Bharu Plot 3, PMP1: Pasir Mas Plot 1, PMP2: Pasir Mas Plot 2, PMP3: Pasir Mas Plot 3, PPP1: Pasir Puteh Plot 1, PPP2: Pasir Puteh Plot 2, and PPP3: Pasir Puteh Plot 3.

**Figure 6:**
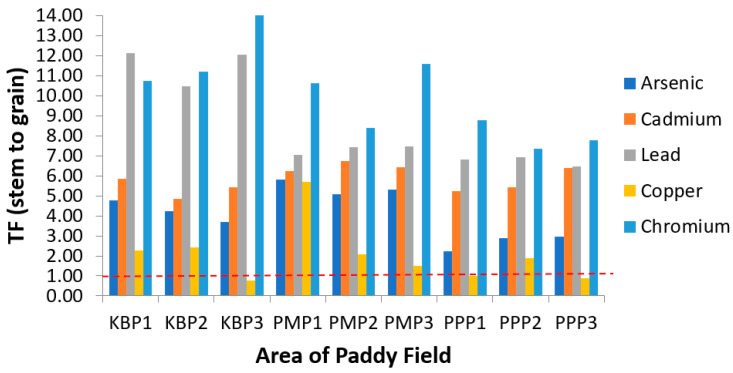
Translocation factor of the heavy metals from stem to grain (TF_Stem_) at different sampling sites. KBP1: Kota Bharu Plot 1, KBP2: Kota Bharu Plot 2, KBP3: Kota Bharu Plot 3, PMP1: Pasir Mas Plot 1, PMP2: Pasir Mas Plot 2, PMP3: Pasir Mas Plot 3, PPP1: Pasir Puteh Plot 1, PPP2: Pasir Puteh Plot 2, and PPP3: Pasir Puteh Plot 3.

Translocation factor (TF) describes metal transferability from soil to plant and demonstrates the mobility and accumulation of heavy metals in the upper parts of plant [52]. Greater TF values of heavy metals indicate their higher mobility or availability in the plants [66,67,68]. The TF values of studied metals for soil to root (TF_Soil_), root to stem (TF_Root_) and stem to grain (TF_Stem_) are presented in Figure 4, Figure 5 and Figure 6, respectively. The ranking order of TF_Soil_ was Cu > As > Pb > Cd > Cr. According to Figure 5, TF_Root_ in general is below 1 for all sites and metals, with only some exceptions. Meanwhile, the translocation values of the metals from stem to grain (TF_Stem_) presented in Figure 6 show all the metals exceeded 1 in the descending order Cr > Pb > Cd > Cu > As. It has been reported, plant factors significantly influence Cu uptake from soil by roots, and diverse plants are characterised by different root activities and exudates that influence the solubility and phytoavailability of Cu in soil [23]. The TF_Soil_ value for Cd obtained in the current study contradicts previous studies where Cd was maximal (TF_Soil_ > 1), and Cd(II) is easily released from the soil in comparison with other hazardous cations [32,66,68]. The extensive accumulation of Cd^2+^ in paddy roots indicates that Cd^2+^ is more bioavailable to plants in comparison with the other heavy metals, leading to a higher biological absorption coefficient [32]. Therefore, as illustrated in the current study, Cd has poor bioavailability from soil to root due to the TF_Soil_ being <1. The TF_Root_ for Cu in PMP3 was greater than 1 with a value of 1.70. The maximal value of TF_Root_ for Cu may be due to the development of highly specific mechanisms of translocation and storage for micronutrients. The same mechanisms are also participating in the uptake, translocation and storage of harmful elements, whose chemical properties are similar to those of essential elements [69,70].

In the case of Cd, the TF_Root_ in PPP1 was also >1 which indicates Cd translocated effectively from root to stem which concurs with its known ability to readily translocate in plants [71]. Cr and Pb fall in the lowest category for translocation capacity to plant shoots [72]. The TF_Stem_ for Cr and Pb were >1 in accordance with published work and indicates that *Oryza sativa* effectively translocates Cr and Pb from stem to grain [73]. The high values of TF_Stem_ for Cr and Pb may be due to the high level of these metals in soil samples. The value of TF_Stem_ was higher than TF_Root_ and TF_Soil_, which is inconsistent with previous reports where the TF of grain was lower than those of root and stem for heavy metals [32]. This demonstrates that the mobility of heavy metals from stem to grain was extremely efficient and large amount of heavy metals were transferred to the paddy grain. As a result, consumption of such rice as staple food probably threatens the health of the public. The process of metal translocation is a key factor affecting the metals distribution in different parts of the plant [74]. Hence, the pattern of metal translocation from root to other parts of the plant may be a valuable indicator in assessing heavy metals contamination [25,32]. Anatomical, biochemical and physiological factors have been identified that contribute to the distribution and storage of heavy metal in the upper parts of plants [75].

### 3.6. Health Risk Assessment (HRA) of Heavy Metals

Consumption of rice is recognised as a significant route of exposure to toxic heavy metals in humans [29]. The overall results show that the average daily dose (ADD) of studied heavy metals for both children and adults did not exceed the safe intake limit, though the values for children were relatively higher than those for adults. This is probably due to the larger body weight of adults. However, this finding does not lend support to previous reports in which the dietary intake of heavy metals was higher in adults due to larger amounts of rice consumption, as compared with children [29,38]. A possible explanation for the difference is that the values for body weight and ingestion rate used for the ADD calculations differ between current and previous studies. Table 8 shows the ADD of heavy metals for adults and children via rice consumption from Kelantan. In both adults and children, the heavy metals with the lowest ADD values was Cd. The safe intake limits of As, Cd and Pb for both adults and children are 0.003, 0.0008 and 0.0015 mg/kg/day, respectively [76,77]. Based on Dietary Reference Intakes (DRIs) from National Academies, the safe intake limit of adults for Cu and Cr are in the ranges of 0.7 to 0.9 and 0.020 to 0.035 mg/kg/day, respectively, while for children the safe intake limits for Cu and Cr are between 0.340 to 0.440 and 0.011 to 0.015 mg/kg/day, respectively.

**Figure 7:**
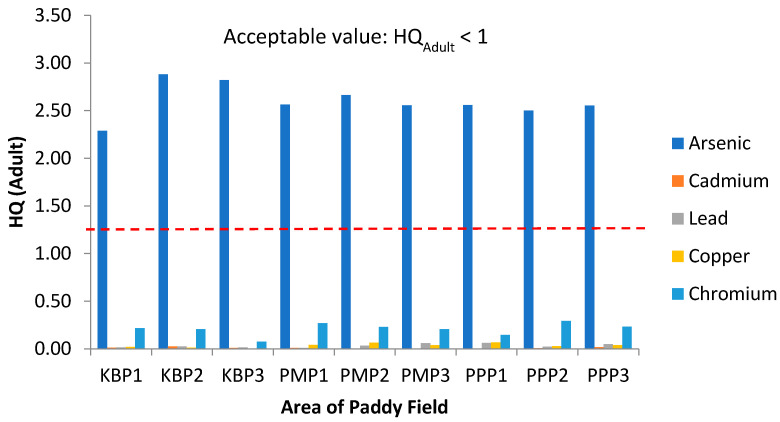
Hazard Quotient (HQ) values for adults via intake of rice from the three plots of sampling sites. KBP1: Kota Bharu Plot 1, KBP2: Kota Bharu Plot 2, KBP3: Kota Bharu Plot 3, PMP1: Pasir Mas Plot 1, PMP2: Pasir Mas Plot 2, PMP3: Pasir Mas Plot 3, PPP1: Pasir Puteh Plot 1, PPP2: Pasir Puteh Plot 2, and PPP3: Pasir Puteh Plot 3.

**Figure 8:**
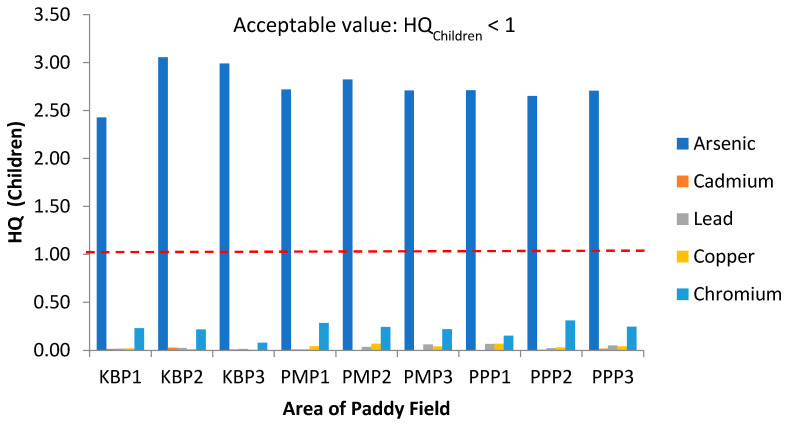
Hazard Quotient (HQ) values for children via intake of rice from the three plots of sampling sites. KBP1: Kota Bharu Plot 1, KBP2: Kota Bharu Plot 2, KBP3: Kota Bharu Plot 3, PMP1: Pasir Mas Plot 1, PMP2: Pasir Mas Plot 2, PMP3: Pasir Mas Plot 3, PPP1: Pasir Puteh Plot 1, PPP2: Pasir Puteh Plot 2, and PPP3: Pasir Puteh Plot 3.

To assess the non-carcinogenic health risk, the hazard quotient (HQ) values for individual heavy metals in adults and children are displayed in Figure 7 and Figure 8, respectively. All HQ values of As for both adults and children were greater than 1 (HQ >1), which is above the acceptable value. Arsenic possessed the highest HQ in all studied areas. The ranking order of HQ for adults was As > Cr > Cu > Pb > Cd. Similarly, for children, the same trend was depicted. HQ values obtained in this study revealed the presence of non-carcinogenic risk in local residents (both adults and children) due to exposure to As through rice consumption. It is pertinent to point out that this observation is not consistent with a previously published study where the HQ values for nine heavy metals including Al, Cd, Cu, Co, Cr, Fe, Zn, As and Pb, were HQ <1. The difference may be ascribed due to different types of rice were used for analysis where commercial rice instead of a raw rice grain from paddy fields [78].

**Figure 9:**
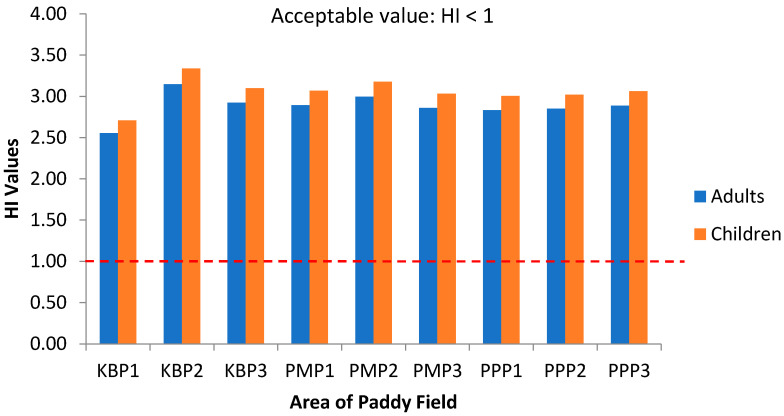
Hazard Index (HI) values of adults and children via intake of rice from the three plots of sampling sites. KBP1: Kota Bharu Plot 1, KBP2: Kota Bharu Plot 2, KBP3: Kota Bharu Plot 3, PMP1: Pasir Mas Plot 1, PMP2: Pasir Mas Plot 2, PMP3: Pasir Mas Plot 3, PPP1: Pasir Puteh Plot 1, PPP2: Pasir Puteh Plot 2, and PPP3: Pasir Puteh Plot 3.

The hazard index (HI) values are depicted in Figure 9 as combined exposure to heavy metals via rice consumption for three plots of Kota Bharu, Pasir Mas and Pasir Puteh. The combined HI values for all five heavy metals exceeded the acceptable value (HI > 1). Children are more prone to the non-carcinogenic health risks since the HI values were slightly higher compared with the adult in all studied areas and may be attributed to the high ADD values of children. In contrast, in a previous study, it was reported that adults are more vulnerable to non-carcinogenic health risks because of the higher HI values compared with children [78]. Hence, the perennial consumption of contaminated rice grain by local inhabitants possibly constitutes a relatively high risk to health.

**Figure 10:**
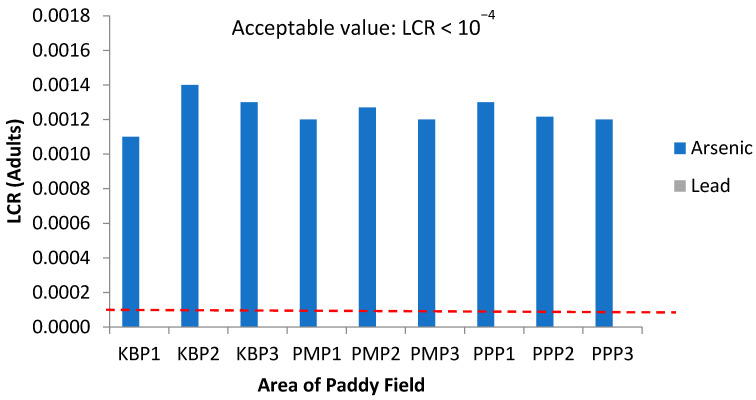
Lifetime cancer risk (LCR) values for adults via intake of rice from the different sampling sites in Kelantan. KBP1: Kota Bharu Plot 1, KBP2: Kota Bharu Plot 2, KBP3: Kota Bharu Plot 3, PMP1: Pasir Mas Plot 1, PMP2: Pasir Mas Plot 2, PMP3: Pasir Mas Plot 3, PPP1: Pasir Puteh Plot 1, PPP2: Pasir Puteh Plot 2, and PPP3: Pasir Puteh Plot 3.

**Figure 11:**
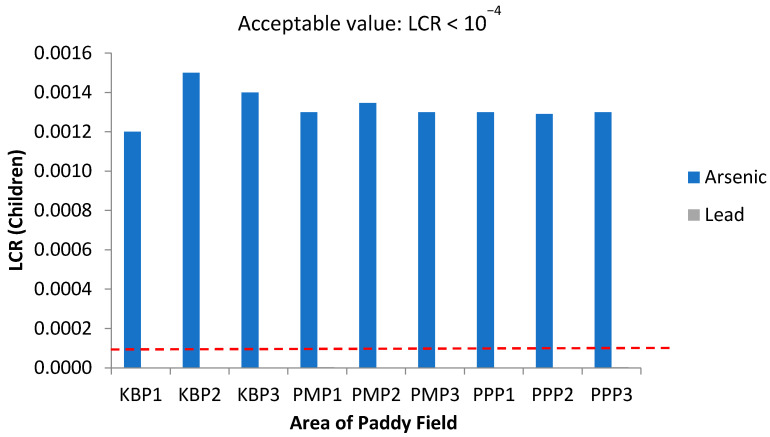
Lifetime cancer risk (LCR) values for children via intake of rice from the different sampling sites in Kelantan. KBP1: Kota Bharu Plot 1, KBP2: Kota Bharu Plot 2, KBP3: Kota Bharu Plot 3, PMP1: Pasir Mas Plot 1, PMP2: Pasir Mas Plot 2, PMP3: Pasir Mas Plot 3, PPP1: Pasir Puteh Plot 1, PPP2: Pasir Puteh Plot 2, and PPP3: Pasir Puteh Plot 3.

**Figure 12:**
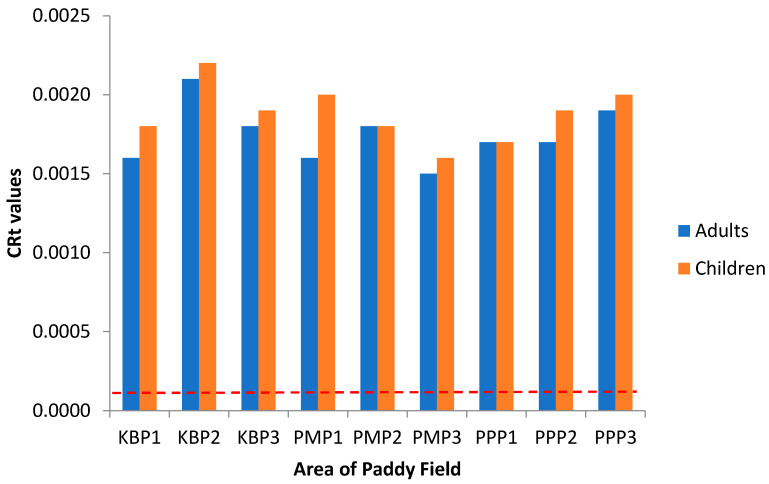
The total cancer risk (CRt) for adults and children via intake of rice from the different sampling sites in Kelantan. KBP1: Kota Bharu Plot 1, KBP2: Kota Bharu Plot 2, KBP3: Kota Bharu Plot 3, PMP1: Pasir Mas Plot 1, PMP2: Pasir Mas Plot 2, PMP3: Pasir Mas Plot 3, PPP1: Pasir Puteh Plot 1, PPP2: Pasir Puteh Plot 2, and PPP3: Pasir Puteh Plot 3.

Concerning carcinogenic risk, Figure 10 and Figure 11 present the lifetime cancer risk (LCR) values of heavy metals via the intake of rice for adults and children in the studied regions, since rice is taken as staple food by local residents. The LCR values for Pb in all sampling sites are within the acceptable limit (LCR < 10^−^^4^), in accordance with [78], while those of As are above the acceptable limit (LCR > 10^−^^4^), in line with [79], indicating a potential carcinogenic risk through rice consumption. The high LCR value for As indicates its presence in inorganic form and high bioaccessibility for the consumer [80]. Nevertheless, there is the possibility of overestimation of this carcinogenic risk of As since As in food does not totally present as the inorganic form [81]. Figure 12 shows the total cancer risk (CRt) resulting from combined exposure to multiple carcinogenic heavy metals in the rice grain collected from Kelantan. For carcinogenic risk assessment, the acceptable range recommended by USEPA are 10^−6^ to 10^−4^, which means that the probability of one to one hundred in a million in developing cancer during the course of a 70-year lifetime is considered as an acceptable or insignificant risk. The CRt values for all studied areas, and for both adults and children, were greater than 10^−4^, revealing a high potential carcinogenic risk due to rice consumption. This is in accordance with findings from previous studies whereby the simultaneous occurrence of heavy metals in rice leads to potential carcinogenic risk [78,82], and it is approximately 81.2% of the cancer risk attributed to Cd. In this study, the LCR values for As have contributed extensively to the CRt value, from which it may be inferred that the cancer risk may largely be attributed to this metal rather than to Cd. The CRt values of children, for all studied areas, were relatively higher than those of adults; metals such as As and Pb can cause negative impacts in the brain development in children [27]. The present study focused solely on the intake of toxic heavy metals through rice consumption. However, people can also be exposed to these toxic contaminants through other contaminated food commodities, including vegetables, fruits, fish, meat and dairy, while water source contamination, dust inhalation and skin contact may also cause human exposure [43,83].

## 4. Conclusions

In sum, the concentration of As, Cd, Pb, Cu and Cr in paddy soils and plants collected from the eastern region of Malaysia was below the maximum allowable limit recommended by relevant standard and regulation, which revealed that the soils and rice grain were safe for agriculture and consumption, respectively. However, accumulation of some heavy metals such as As and Cr in rice grain were found, which may eventually result in negative health effects. The value of enrichment factors (EF) and translocation factors (TF) of heavy metals from soil to shoots of paddy plants were quantified in this study, and the high mobility transfer from stem to rice grain should be a concern to avoid the great accumulation of heavy metals in rice grain since it is taken as staple food. By conducting health risk assessment from rice consumption, non-carcinogenic and carcinogenic risks were likely to present in both local adults and children, with As exposure as the major contributor. This finding suggested that effective measures should be implemented to monitor the heavy metal contamination in this region and to reduce the health risk due to heavy metals exposure, especially in children whose growth and development could be disrupted by the toxic elements. As an alternative to reduce the contamination level of toxic metals as well as to reduce the health risk in the local population, the organic application can be used in paddy cultivation. Moreover, the findings of this present study were obtained using raw rice grain, which were not processed as the rice commercially available. Therefore, as for further exploration, the health risk assessment can be further extended to rice grain available in market, since the study findings can be influenced by the variation, level and distribution of heavy metal in rice grain cultivated. Lastly, synergism and antagonism between the heavy metals of concern in the paddy soils and plants, as well as with other common environmental toxicants, such as polycyclic aromatic hydrocarbons (PAHs), can be further studied to provide deeper understanding regarding their toxicity effect and mechanism in the rice consumers.

## Figures and Tables

**Table 1:** Reference doses (RfD) of five heavy metals.

Elements	RfD (mg/kg/day)	Source
Cd	1.00 × 10^−3^	IRIS ^a^
Cr	1.5	IRIS
Cu	4.00 × 10^−2^	IRIS
As	3.00 × 10^−4^	IRIS
Pb	3.6 × 10^−3 c^	WHO ^b^

Note: ^a^ Integrated Risk Information System, U.S. EPA; ^b^ World Health Organization, WHO; ^c^ Calculated according to the provisional tolerable weekly intake, 25 µg/kg BW, WHO/FAO (1999).

**Table 2:** Concentration of heavy metals in paddy soils collected from Kelantan.

Plot	Concentration of Heavy Metals in Paddy Soil (mg/kg)				
	Arsenic (As)	Cadmium (Cd)	Lead (Pb)	Copper (Cu)	Chromium (Cr)
**KBP**	0.265 ± 0.04 ^c^	0.012 ± 0.000 ^a^	0.423 ± 0.136 ^a^	0.344 ± 0.033 ^ab^	0.146 ± 0.015 ^a^
**PMP**	0.429 ± 0.03 ^b^	0.012 ± 0.000 ^a^	0.223 ± 0.046 ^a^	0.381 ± 0.055 ^a^	0.193 ± 0.029 ^a^
**PPP**	0.544 ± 0.02 ^a^	0.012 ± 0.000 ^a^	0.480 ± 0.210 ^a^	0.260 ± 0.024 ^b^	0.155 ± 0.007 ^a^

Note: Results are presented as Mean ± SD for the concentration of heavy metals in paddy soils collected from Kelantan. ^a–c^ Values with different superscripts in the columns refer to statistically significant difference (*p* < 0.05) between different sampling areas. KBP: Kota Bharu Plot, PMP: Pasir Mas Plot, PPP: Pasir Puteh Plot.

**Table 3:** Concentration of arsenic in different parts of paddy plants collected from Kelantan.

Plot	Concentration of Arsenic (As) (mg/kg)			
	Root	Stem	Leaf	Grain
**KBP**	0.079 ± 0.001 ^a^	0.125 ± 0.035 ^a^	0.021 ± 0.001 ^b^	0.081 ± 0.004 ^a^
**PMP**	0.071 ± 0.006 ^a^	0.039 ± 0.005 ^b^	0.029 ± 0.003 ^b^	0.087 ± 0.005 ^a^
**PPP**	0.078 ± 0.001 ^a^	0.079 ± 0.003 ^ab^	0.147 ± 0.008 ^a^	0.087 ± 0.002 ^a^

Note: Results are presented as Mean ± SD for concentration of arsenic in different parts of paddy plants collected from Kelantan. ^a^^–b^ Values with different superscripts in the columns refer to statistically significant difference (*p* < 0.05) between different sampling areas. KBP: Kota Bharu Plot, PMP: Pasir Mas Plot, PPP: Pasir Puteh Plot.

**Table 4:** Concentration of cadmium in different parts of paddy plants collected from Kelantan.

Plot	Concentration of Cadmium (Cd) (mg/kg)			
	Root	Stem	Leaf	Grain
**KBP**	0.002 ± 0.001 ^a^	0.003 ± 0.002 ^a^	0.001 ± 0.000 ^a^	0.002 ± 0.001 ^a^
**PMP**	0.001 ± 0.000 ^a^	0.009 ± 0.005 ^a^	0.013 ± 0.004 ^a^	0.000 ± 0.000 ^a^
**PPP**	0.001 ± 0.000 ^a^	0.002 ± 0.000 ^a^	0.013 ± 0.012 ^a^	0.001 ± 0.000 ^a^

Note: Results are presented as Mean ± SD for concentration of cadmium in different parts of paddy plants collected from Kelantan. ^a^ Values with different superscripts in the columns refer to statistically significant difference (*p* < 0.05) between different sampling areas. KBP: Kota Bharu Plot, PMP: Pasir Mas Plot, PPP: Pasir Puteh Plot.

**Table 5:** Concentration of lead in different parts of paddy plants collected from Kelantan.

Plot	Concentration of Lead (Pb) (mg/kg)			
	Root	Stem	Leaf	Grain
**KBP**	0.007 ± 0.001 ^a^	0.037 ± 0.016 ^a^	0.016 ± 0.006 ^ab^	0.007 ± 0.001 ^a^
**PMP**	0.004 ± 0.003 ^a^	0.003 ± 0.000 ^b^	0.007 ± 0.005 ^b^	0.005 ± 0.004 ^a^
**PPP**	0.011 ± 0.005 ^a^	0.011 ± 0.001 ^b^	0.040 ± 0.017 ^a^	0.013 ± 0.005 ^a^

Note: Results are presented as Mean ± SD for concentration of lead in different parts of paddy plants collected from Kelantan. ^a–b^ Values with different superscripts in the columns refer to statistically significant difference (*p* < 0.05) between different sampling areas. KBP: Kota Bharu Plot, PMP: Pasir Mas Plot, PPP: Pasir Puteh Plot.

**Table 6:** Concentration of copper in different parts of paddy plants collected from Kelantan.

Plot	Concentration of Copper (Cu) (mg/kg)			
	Root	Stem	Leaf	Grain
**KBP**	0.052 ± 0.045 ^a^	0.573 ± 0.866 ^a^	0.628 ± 0.391 ^a^	0.015 ± 0.008 ^b^
**PMP**	0.035 ± 0.003 ^a^	0.066 ± 0.009 ^a^	0.043 ± 0.022 ^b^	0.044 ± 0.001 ^a^
**PPP**	0.032 ± 0.007 ^a^	0.030 ± 0.005 ^a^	0.048 ± 0.010 ^b^	0.032 ± 0.008 ^a^

Note: Results are presented as Mean ± SD for concentration of copper in different parts of paddy plants collected from Kelantan. ^a–b^ Values with different superscripts in the columns refer to statistically significant difference (*p* < 0.05) between different sampling areas. KBP: Kota Bharu Plot, PMP: Pasir Mas Plot, PPP: Pasir Puteh Plot.

**Table 7:** Concentration of chromium in different parts of paddy plants collected from Kelantan.

Plot	Concentration of Chromium (Cr) (mg/kg)			
	Root	Stem	Leaf	Grain
**KBP**	0.075 ± 0.006 ^a^	0.055 ± 0.016 ^a^	0.070 ± 0.010 ^a^	0.068 ± 0.002 ^a^
**PMP**	0.070 ± 0.019 ^a^	0.078 ± 0.007 ^a^	0.020 ± 0.013 ^b^	0.086 ± 0.015 ^a^
**PPP**	0.080 ± 0.006 ^a^	0.052 ± 0.006 ^a^	0.081 ± 0.026 ^a^	0.082 ± 0.005 ^a^

Note: Results are presented as Mean ± SD for concentration of Chromium in different parts of paddy plants collected from Kelantan. ^a–b^ Values with different superscripts in the columns refer to statistically significant difference (*p* < 0.05) between different sampling areas. KBP: Kota Bharu Plot, PMP: Pasir Mas Plot, PPP: Pasir Puteh Plot.

**Table 8:** Average daily dose (ADD) of heavy metals for adults and children via rice consumption.

**Metals**	**ADD (Adults) (mg/kg/day)**									**Safe Value (mg/kg/day)**
	**KBP1**	**KBP2**	**KBP3**	**PMP1**	**PMP2**	**PMP3**	**PPP1**	**PPP2**	**PPP3**	
As	0.0009	0.00086	0.00085	0.00077	0.00080	0.00077	0.00077	0.00079	0.00077	0.003
Cd	0.00001	0.00002	0.00001	0.00001	0.00000	0.00000	0.00000	0.00001	0.00002	0.0008
Pb	0.00005	0.00009	0.00005	0.00003	0.00012	0.00021	0.00022	0.00008	0.00017	0.0015
Cu	0.00019	0.00012	0.00005	0.00042	0.00065	0.00038	0.00066	0.00029	0.00038	0.7–0.9
Cr	0.00065	0.00062	0.00076	0.00081	0.00069	0.00062	0.00043	0.00084	0.00070	0.020–0.035
**Metals**	**ADD (Children) (mg/kg/day)**									**Safe Value (mg/kg/day)**
	**KBP1**	**KBP2**	**KBP3**	**PMP1**	**PMP2**	**PMP3**	**PPP1**	**PPP2**	**PPP3**	
As	0.00096	0.00092	0.00090	0.00082	0.00085	0.00081	0.00081	0.00083	0.00081	0.003
Cd	0.00001	0.00003	0.00001	0.00001	0.00000	0.00000	0.00000	0.00001	0.00002	0.0008
Pb	0.00006	0.00009	0.00005	0.00003	0.00013	0.00022	0.00024	0.00008	0.00018	0.0015
Cu	0.00020	0.00013	0.00005	0.00044	0.00068	0.00040	0.00070	0.00031	0.00041	0.340–0.440
Cr	0.00069	0.00065	0.00081	0.00086	0.00073	0.00066	0.00046	0.00089	0.00074	0.011–0.015

Note: KBP1: Kota Bharu Plot 1, KBP2: Kota Bharu Plot 2, KBP3: Kota Bharu Plot 3, PMP1: Pasir Mas Plot 1, PMP2: Pasir Mas Plot 2, PMP3: Pasir Mas Plot 3, PPP1: Pasir Puteh Plot 1, PPP2: Pasir Puteh Plot 2, and PPP3: Pasir Puteh Plot 3.

## Data Availability

Data is contained within the article.
